# Geochemical characterization and assessment of fluoride sources in groundwater of Siloam area, Limpopo Province, South Africa

**DOI:** 10.1038/s41598-021-93385-4

**Published:** 2021-07-07

**Authors:** Tobiloba Onipe, Joshua N. Edokpayi, John O. Odiyo

**Affiliations:** 1 Department of Hydrology and Water Resources, University of Venda, Private bag X5050, Thohoyandou, 0950 South Africa; 2 DVC: RICl, Vaal University of Technology, Vanderbiljpark, South Africa

**Keywords:** Environmental sciences, Chemistry

## Abstract

Siloam’s groundwater is reportedly characterized by high fluoride. In response to the reported high incidence of dental fluorosis in the area, sources of elevated fluoride in the groundwater were investigated. Total fluoride (TF) was determined using Ion Chromatograph and Fluoride Ion Selective Electrode. The mineral composition of rocks and soils were determined using X-ray Fluorescence and X-ray diffraction, respectively. Results revealed that groundwater fluoride concentration ranged from 3.92 to 4.95 mg/L. Na-Cl water type was found to be dominant in the water samples. TF content of the rocks and soils ranged from 10 to 2000 mg/L. Leachates were obtained by making a slurry from the samples at a predetermined temperature and time. TF in leachates ranged between 0.27 and 14.88 mg/L and 0.05 to 10.40 mg/L at induced, and non-induced temperatures, respectively. The possible source of fluoride has been previously inferred to be caused by fluorite minerals occurring at greater depth. However, this study proves that fluoride decreases with depth and the elevated fluoride in the groundwater is caused by smectite-kaolinite clay, muscovite and chlorite minerals abundant in the area. Geothermal temperature exhibited by the groundwater in the area is a major factor enhancing the release of fluoride from the clay materials.

## Introduction

Water is an important basic resource necessary for human development and economic growth. The supply of clean and safe water over the last decade has improved progressively globally^1^. Despite this recorded success, millions of people are not connected to water supply infrastructure and often resort to several alternative sources for their domestic water needs^2,3^. One of such sources include the exploitation of groundwater^4^.

Groundwater is the largest deposit of freshwater on earth and is a dependable alternative source of water with more than 75% of African population depending on it for survival^5^. However, there are various misconceptions about groundwater that it is free from chemical contamination and pathogens owing to its aesthetic property. Over the years, studies have shown that groundwater is exposed to various chemical contaminants from natural geological processes and anthropogenic activities^6–8^. Groundwater are often exploited in the form of natural springs, wells and drilling of boreholes.

Several contaminants have been reported in the groundwater which are of potential risk to public health. Amongst which are the presence of elevated levels of arsenic, lead, cadmium, mercury, fluoride, nutrients and microorganisms^9–11^. Some of these contaminants are more linked to geogenic sources than others. Fluoride is believed to be caused majorly by fluoride bearing minerals with little or no contribution from human activities. However, groundwater contamination from lead, nutrients and microorganisms are more linked to anthropogenic activities such as mining and agriculture^10,11^.

Fluoride is very important in the healthy development of the skeletal and dental framework of the body, if present in levels < 0.5 mg/L, can lead to dental caries while concentration exceeding 1.5 mg/L can lead to fluorosis and no fluorosis diseases. The levels of fluoride vary globally and various climatic, hydrological and geochemical properties often determine and influence its levels in groundwater. The consumption of fluoride rich water has been linked to various public health burden notable among which are dental and skeletal fluorosis^12–14^. Cases of several non-fluorosis diseases such as Alzheimer’s disease, loss of mobility, infertility, hearing difficulty, retarded growth, low intelligence quotient and cancer have also been linked to the consumption of fluoride rich water^9,15–20^. Occurrence of elevated fluoride in groundwater is an emerging world-wide threat that affects over 200 million people worldwide and over 80 million people in East Africa^21^.

The occurrence of high levels of fluoride exceeding the permissible level of 1.5 mg/L by the World Health Organization has been recorded in many regions of the world. The East Africa rift valley system have been renowned for high levels of fluoride in groundwater^22–24^. In most parts of South Africa, elevated levels of fluoride have been reported in groundwater. The Northern region of the Limpopo Province which is semi-arid consist of numerous springs which are also known to contain high fluoride concentration^9,25,26^.

Globally, several efforts have been channeled towards the monitoring of groundwater quality for compliance to regulatory standard due to the number of people that depends on it for survival^27–29^. Fluoride compliance study have been greatly reported in literature^7,8,26^. However, very few studies have reported the source of fluoride in groundwater^9,18,25^. The most important factor controlling the occurrence of fluoride naturally in groundwater is the rock types (geology) of the area. Fluorine has been reported in all the major rock types (igneous, sedimentary and metamorphic)^9,18^. Notable geogenic sources that have been reported include fluoride bearing minerals such as fluorite, villiaumite, apatite, biotite, amphibole, micas, topaz, cryolite, muscovite and fluorspar^9,18,25,30,31^.

In Siloam and the Northern part of South Africa, studies from our research group and other researchers have shown the occurrence of elevated fluoride concentrations in wells, boreholes (cold and hot) and geothermal springs^25,26,31–34^. Siloam area is known for a large-scale occurrence of dental fluorosis amongst children and adults and according to Odiyo and Makungo^35^, over 85% of the population in the study area could be affected by dental fluorosis. The groundwater chemistry of this region has not been fully explored or reported. Therefore, in this study, we report mainly on the occurrence of fluoride bearing minerals in the region and some factors that could favor its dissolution.

## Description of the study area

### Location

**Figure 1:**
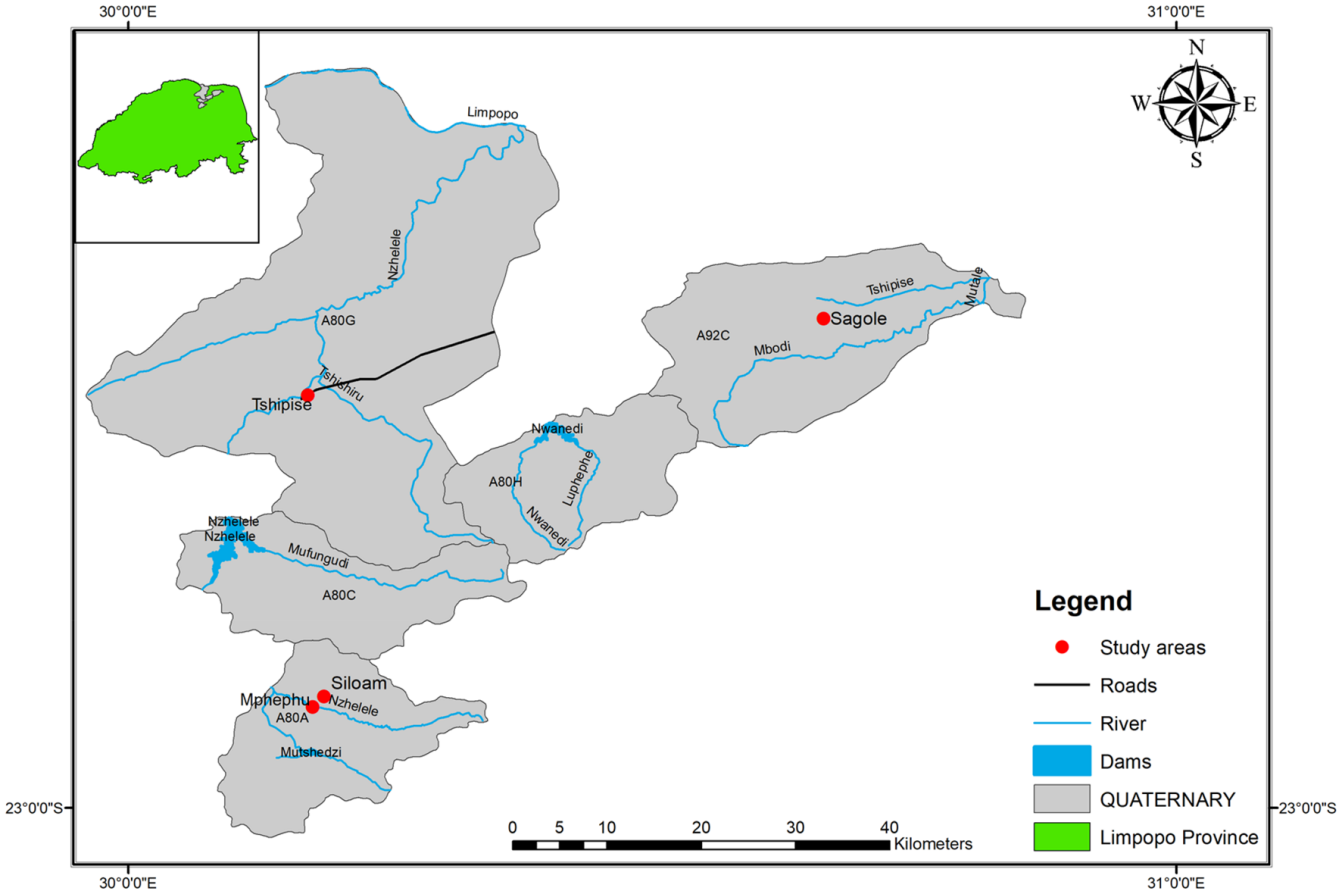
Map of the study area (Figure drawn using ArcGIS version 10.4, licensed to the University of Venda, South Africa).

The study area (Fig. 1) is located within Makhado Municipality, Vhembe District in Limpopo Province of South Africa. This includes the northern flank of Tswime Mountain where Mphephu thermal spring is located. It is 60 km northeast of Makhado and is approximately 45 km west of Thohoyandou. Siloam falls under surface water and groundwater quaternary catchment A80A of the Nzhelele River catchment, which is in the northern region of Limpopo Province, South Africa^36^. The Nzhelele River flows in the northwest direction, towards Nzhelele Dam^26^. The prevailing seasons are winter and summer, with average winter temperatures ranging between 16 and 22 °C and average summer temperatures varies between 22 to 40 °C^37^. Siloam experiences seasonal rainfall, ranging from 350 – 400 mm per annum in the summer months of September to March^36,38^. Rainfall in Siloam is largely influenced by its position on the leeward side of Soutpansberg mountain which plays an important role in the groundwater recharge of the area^35^. Evaporation rate is higher than the precipitation rate at 1300–1400 mm/annum. Siloam is dominated by two major geothermal springs and other hot private and community borehole. Boreholes in the study area varies in depth and are usually within the range of 65–85 m and the mean depth to groundwater is about 15–25 m^33^.

### Geology

**Figure 2:**
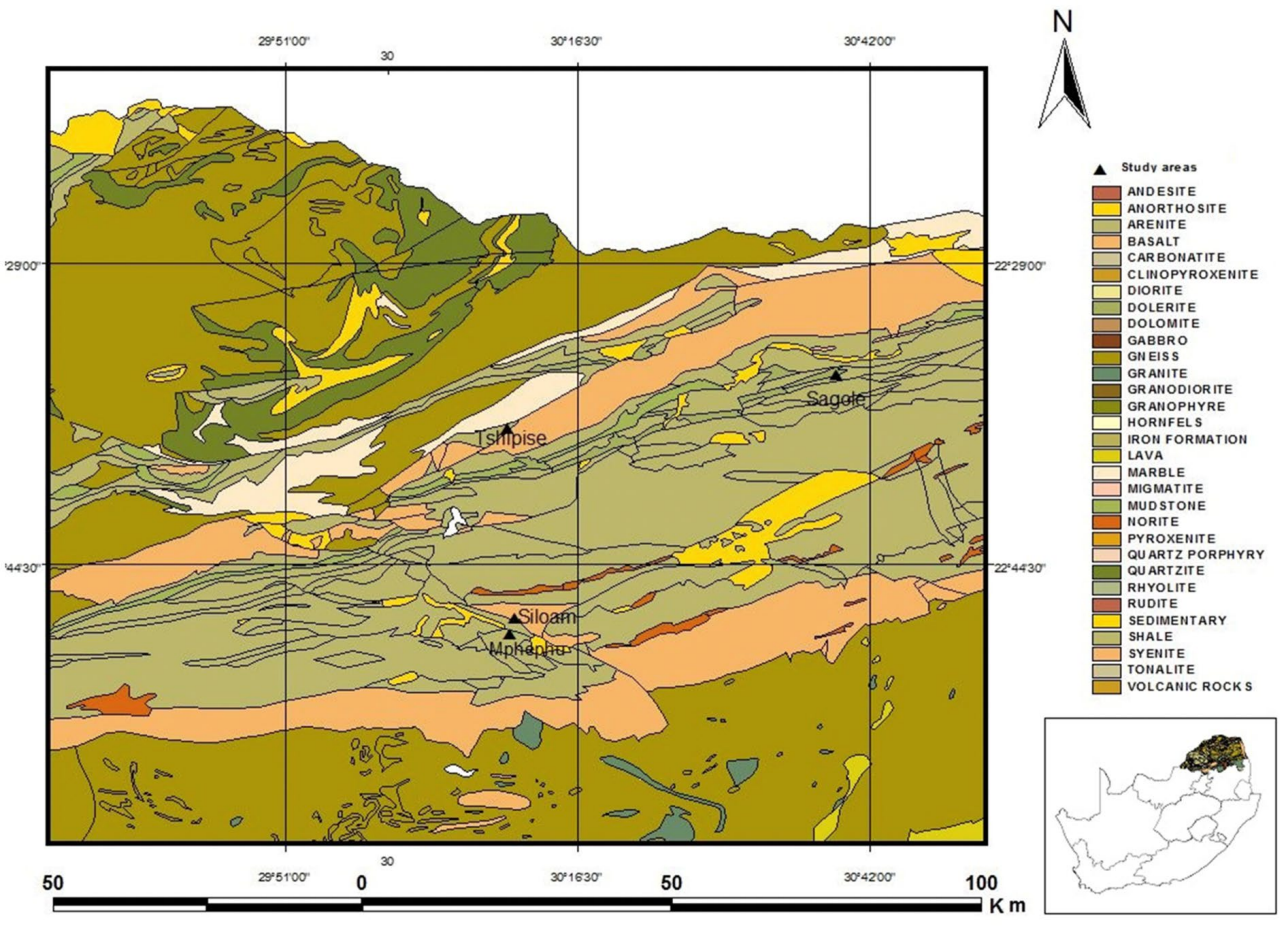
Geological map of the study area^32^, permission granted by Dr. Durowoju).

Siloam lies within the Soutpansberg group (Fig. 2). Soutpansberg group is a Mokolian age extensively faulted succession that provides a link between the pre-and post-Bushveld age (younger than 1800 mya) rocks^39^. The Soutpansberg succession is an east to west trending asymmetrical rift overlying the Palala shear belt^39^. Soutpansberg group is subdivided into six formations namely: Tshifhefhe formation, Sibasa formation, Fundudzi formation, Wyllie’s formation, Musekwa formation and Nzhelele formation^40,41^. The Palala shear belt separates the Kaapval craton in the South and the Limpopo belt in the North^39^. The area has been through different post-deposition tectonic and erosional activities. The evolution of Soutpansberg started with the deposition of basaltic lava as a result of volcanic activities, followed by the deposition of syn-rift sequence of sedimentary rock. The area was subjected to an extensive erosional period of non-deposition after which resistant sandstone-quartzite was deposited^42^. Unconformable deposition of Karoo supergroup sediments occurred and altogether, went through the process of block-faulting^43^. The sequence is best developed from north of Siloam fault to Musekwa mountains^44^.

## Materials and methods

### Sampling of soil, water and rock

One soil and rock samples were collected from the surface in the study area while twelve borehole cuttings from the subsurface were sampled for analysis. Four water samples were collected across the area. The samples were collected using plastic cups as recommended by Harvey^45^. For each location, the samples were collected three times in a 1 L sampling bottle and were transported to the laboratory for analysis. As suggested by Weaver et al.^46^, the bottles were pre-rinsed with the water to be sampled, to avoid cross contamination. The groundwater samples for anion analysis were preserved in a refrigerator at a temperature of 4 °C and analyzed within seven days of collection. However, the samples for cation and trace metals analysis were preserved with concentrated nitric acid. Table 1 below summarizes the sample types and number.

**Table 1:** Description of Sample types.

Sample type	Sample ID	Depth (m)	Location	No of samples
**Water samples**				
Monitoring borehole 1	BH1	> 60	22° 53′ 34.008″ S 30° 11′ 47.22″ E	1
Monitoring borehole 2	BH2	> 60	22° 53′ 38.148″ S 30° 11′ 18.52″ E	1
Private Borehole 1	H1	> 60	22° 53′ 39.43" S 30° 11′ 43.94" E	1
Private Borehole 2	H2	> 60	22°53′43.39"S 30°11′32.98"E	1
**Rocks and soil samples**				
Surface Soil Sample	S1	N/A	22° 53′ 34.83″ S 30° 11′ 45.56″ E	1
Surface Rock Sample 1	SR2	N/A	22° 54′ 22.8″ S 30° 10′ 44.0″ E	1
Surface Rock Sample 2	SR3	N/A	22° 52′ 02.5″ S 30° 12′ 09.7″ E	1
Borehole 1 Cuttings	X	1–65	22° 53′ 34.008″ S 30° 11′ 47.22″ E	6
Borehole 2 Cuttings	Y	1–40	22° 53′ 38.148″ S 30° 11′ 18.528″ E	6

*N/A* not available.

### Measurement of basic water quality parameters

The pH, electrical conductivity (EC) and temperature were measured *insitu* using a multimeter (Multi 340i/SET). The instrument was calibrated prior to measurement in adherence to the manufacturer’s guideline.

### Major anions analysis

Groundwater samples were analyzed for major anions content (fluoride, nitrate, phosphate, bicarbonate) using an Ion Chromatograph (Dionex Model DX 500). Prior to analysis, the samples were filtered using 0.45 μm syringe filter. The samples were placed in vails associated with an auto sampler^1^. The EC of the samples were below 500 µs/cm hence dilution was not done.

### Trace elements analysis for groundwater and rock samples

Major cations in the acidified groundwater water samples were analyzed using Inductively coupled plasma optical emission spectrophotometer (ICP-OES) while Inductively coupled plasma mass spectrophotometer (ICP-MS) were used for the analysis of trace metals. Similarly, Trace and major metals in the soil and rock samples were analyzed using the same analytical instruments outlined above after microwave digestion. The procedure reported by Pais and Jones^47^ for soil and rock sample digestion were adhered to. Briefly: The soil/rock samples were digested using a microwave digestion system (SR ISO 11,466: 1999). Approximately 1.0 g of pre-treated samples were digested with 9 mL HNO_3_ and 1 mL H_2_O_2_. The solutions were allowed to stay overnight at room temperature and then, placed in the microwave for 30 min, followed by cooling. The solutions were diluted to 50 mL with distilled water.

### Preparation of soil and rock samples

The rocks and soil samples for X-ray diffractometer (XRD) analyses were prepared according to the standardized PANalytical backloading system, which provides nearly random distribution of the particles, while the samples for X-ray fluorescence (XRF) were prepared as pressed powders^48^. The rock samples were split, washed, dried, crushed and pulverized. Splitting into chips was done by a hydraulic splitter after which the split chip was washed and treated with ultrasound wave to remove any loose impure particles. After washing, the samples were dried overnight in an oven to remove excess water introduced during washing of the samples. A jaw crusher was used to crush the samples which were further reduced to finer grain (< 40 μm in diameter) by pulverizing them for about five (5) min using a Retsch RS 200 miller. The milled samples were transferred to a paper bag and oven dried at 110 °C for 6 h. After oven drying, the samples were further milled to less than 20 µm^48^.

Samples for total fluoride in soil and rocks were prepared by weighing 0.2 g of the samples in a nickel crucible, 2 g of CaCO_3_ and Na_2_O_2_ was added, respectively. The mixture was fused manually to aid leaching, using a low flame bunsen burner in a fume box. Auto fusion was avoided to prevent burning of the nickel crucibles and ensure that Na_2_O_2_ does not get moist. 250 mL plastic beakers were pre-washed in a heated Eco bath using concentrated HCl. The heated crucibles were transferred into 250 mL plastic beakers where 50 mL of de-ionized water and 8 mL of HCl were added^49^. The mixtures were covered with a plastic lid and placed on warm bath until the samples were leached out of the crucibles. The crucibles were washed with de-ionized water to make sure that all the leachate was removed from the crucibles. The solutions were transferred into 100 mL volumetric flask and diluted to mark. Blank was prepared by fusing 2 g of CaCO_3_ and Na_2_O_2_. The fused mixture was transferred into a 250 mL beaker and 100 mL of de-ionized water and 16 mL of concentrated HCl were added^48^. The mixture was allowed to leach out and the crucible was rinsed with distilled water. The leachate was transferred into a 200 mL volumetric flask and filled to mark with de-ionized water. Calibration was carried out using 0.1 ppm, 1 ppm, 10 ppm, 100 ppm and 1000 ppm fluoride standards. The standards, samples and blanks were all mixed in equal proportion with TISAB II in a 1:1 ratio.

Calibration of pH meter was done using buffers 6.00 and 4.00. The Slope was 97.9% which fell within the recommended slope limit of 95 to 105%. The pH of the samples and blanks were adjusted to 6.00 using 50% NaOH and HCl. The pH was adjusted because at pH below 5, hydrogen ions complex a portion of the fluoride ions, forming HF or HF_2_, which cannot be detected by the fluoride electrode^49^. Likewise, in a solution with pH above 9, the electrode responds to hydroxide ion as well as to fluoride ion giving an exaggerated reading. Samples for leachable fluoride in soil and rocks were prepared by mixing 10 g of the sample with 100 mL of distilled water at a ratio of 1:10. The samples were prepared in duplicate to ensure that each sample is represented under induced temperature and room temperature. The first set of samples were shaken in a warm bath at normal room temperature for 6 h^48^. The second set of samples were shaken in a warm bath at a temperature of 42 °C. Because of the clay contents of the sample the mixture was centrifuged for 10 min at a speed of 3000 rpm. The centrifuged samples were decanted and filtered using a 9 cm filter paper. The resultant mixture was then further filtered using a 40 µm syringe filter to ensure that all impurities that could block the IC column were removed. The EC of the samples were below 500 µs/cm hence dilution was not required.

### Lithology and mineral phase identification

The minerals in the rocks and soil samples from Siloam were identified and quantified using X-ray diffraction method of analysis. The samples were analyzed using a PANalytical X’Pert Pro powder diffractometer in θ–θ configuration with an X’Celerator detector and variable divergence and fixed receiving slits with Fe filtered Co-Kα radiation (λ = 1.789 Å). The phases were identified using X’Pert Highscore plus software. The relative phase amounts (weight%) were estimated using the Rietveld method (Autoquan Program). The angles of the peaks were used to identify the mineral phase while the intensities of the peaks indicated the abundance of each mineral^50^.

### Total oxide analysis

The total rock and soil oxides were measured using X-ray Fluorescence analytical method. The ARL Perform'X Sequential XRF instrument with Uniquant software was used for the analyses^51^. The software analysed for all elements in the periodic Table between Na and U, but only elements found above the detection limits were reported^51^. The intensities for all elements were corrected automatically for line interference and absorption effects due to all the other elements using the fundamental parameter method^49^.

### Total fluoride analysis

Total fluoride analysis were performed on the groundwater samples as well as the leached samples from rocks and soil. The groundwater analysis was carried out using Fluoride Ion-Selective electrode Orion Versastar Advanced Electrochemistry meter, while the fluoride in solids were analyzed using Metrohm fluoride meter with reference electrode^48^. Calibration standards 0.1 ppm, 1 ppm, 10 ppm and 100 ppm were used to calibrate the equipment. Orion TISAB II was used as the ionic strength adjustment buffer and the TISAB II was mixed in equal volume of 50 mL with 50 mL of samples and buffer. The TISAB used were pre-mixed with standard at ratio 1:1. Labotec Magnetic stirrer was used to ensure that the mixture of TISAB and sample was thorough and accurate during measurement.

### Leaching experiment

A form of mini-leaching experiment was used to determine the leachability of fluoride and other major cation and anions from the rocks and soil samples. Ion chromatography (IC) method was used for the analysis of both normal samples and the temperature induced samples. The IC was stabilized for 15 min prior to analysis^48^. The eluent used was Dionex AS22 which is made up of 45 mM Na_2_CO_3_ and 1.4 mM NaHCO_3_. The pH, EC and alkalinity of the water, soil and rock samples were pre-measured using Mantech Titrasip Autotitrator to ensure that the unknown samples fall within the recommended pH and EC limit for using Ion chromatograph^1^. The analysis was carried out using Dionex Model DX 500 Ion Chromatograph.

### Statistical analysis

Experimental data were analyzed using the Statistical Package for Social Sciences (SPSS) (IBM Version 22) and Microsoft Excel 2013 (Microsoft Corp., Santa Rosa, CA). Descriptive statistics using tables, charts and graphs were used to present the chemical analysis data of the groundwater. Data recorded from the geochemical processes were subjected to Piper chart to identify the main chemical compositions of the groundwater. Correlation analysis was performed using a suitable package in SPSS.

## Results and discussion

### Fluoride levels in groundwater samples of the study area

Fluoride levels in the groundwater samples ranged between 3.92–4.95. These levels exceeded the South African National standards (SANS) for drinking water and the guidelines (1.5 mg/L) of the^52,53^. The levels recorded can lead to dental and skeletal fluorosis on the consumers of this waters and they are also at risk of non-fluorosis diseases. Previous studies by Odiyo and Makungo^35^ have reported fluoride levels in the range of 1.7–5.6 mg/L. Similarly, a more recent publication by the authors also reported fluoride levels exceeding 1.5 mg/L. Table 2 presents the fluoride levels results of this study and other related studies on groundwater around the study area.

**Table 2:** Mean fluoride, pH, temperature and calcium levels of groundwater in the study area.

Sample code	Groundwater type	Study location	Fluoride (mg/L)	pH	Temperature (^o^C)	Ca (mg/L)	Reference
H1	Borehole	Siloam	4.55 ± 0.00	8.86 ± 0.01	48.00 ± 0.01	3.54 ± 0.01	This study
H2	Borehole	Siloam	4.95 ± 0.01	9.19 ± 0.06	45.00 ± 0.00	0.82 ± 0.00	This study
BH1	Borehole	Siloam	4.50 ± 0.00	8.17 ± 0.00	25.00 ± 0.01	27.80 ± 1.71	This study
BH2	Borehole	Siloam	3.92 ± 0.01	8.10 ± 0.01	27.00 ± 0.00	12.80 ± 0.61	This study
BH3	Borehole	Siloam	5.60 ± 0.85	8.97 ± 0.49	Nr	0.33 ± 0.71	Odiyo and Makungo^33^
BH4	Borehole	Siloam	5.76 ± 0.55	8.26 ± 0.40	Nr	0.08 ± 0.07	Odiyo and Makungo^33^
BH5	Borehole	Siloam	3.37 ± 1.56	7.54 ± 0.18	Nr	0.47 ± 0.46	Odiyo and Makungo^33^
BH6	Borehole	Siloam	1.51 ± 0.12	7.34 ± 0.27	Nr	1.41 ± 1.25	Odiyo and Makungo^33^
BH7	Borehole	Siloam	1.44 ± 0.18	7.17 ± 0.13	Nr	1.04 ± 0.71	Odiyo and Makungo^33^
BH8	Borehole	Siloam	1.49 ± 0.06	7.44 ± 0.44	Nr	1.62 ± 2.43	Odiyo and Makungo^33^
BH9	Borehole	Siloam	5.59 ± 0.16	8.11 ± 0.35	Nr	0.07 ± 0.02	Odiyo and Makungo^33^
BH10	Borehole	Siloam	5.37 ± 0.48	8.62 ± 0.47	Nr	0.07 ± 0.06	Odiyo and Makungo^33^
BH11	Borehole	Siloam	4.25 ± 0.90	7.30 ± 0.30	Nr	2.95 ± 2.58	Odiyo and Makungo^33^
BH12	Borehole	Siloam	2.21 ± 1.24	7.35 ± 0.16	Nr	1.81 ± 1.65	Odiyo and Makungo^33^
BH13	Borehole	Siloam	5.11 ± 0.49	7.44 ± 0.22	Nr	1.30 ± 0.68	Odiyo and Makungo^33^
BH14	Borehole	Siloam	5.10 ± 1.09	8.70 ± 0.33	35.30 ± 1.53	4.40 ± 2.42	Odiyo and Makungo^35^
BH15	Borehole	Siloam	1.70 ± 0.60	6.90 ± 0.01	26.90 ± 0.64	70.40 ± 13.59	Odiyo and Makungo^35^
GS1	Geothermal spring	Siloam	5.50 ± 0.35	8.40 ± 0.15	63.86 ± 18.19	2.40 ± 0.86	Odiyo and Makungo^35^
GS2	Geothermal spring	Siloam	6.51 ± 0.08	9.39 ± 0.06	67.7 ± 1.68	5.69 ± 0.05	Durowoju et al.^32^
GS3	Geothermal spring	Mphepu	3.43 ± 1.25	8.10 ± 0.05	42 ± 1.12	12.05 ± 0.14	Durowoju et al.^32^
GS4	Geothermal spring	Tshipise	5.50 ± 0.36	8.47 ± 0.22	55 ± 2.24	2.81 ± 0.09	Durowoju et al.^32^
GS5	Geothermal spring	Sagole	1.69 ± 0.59	8.38 ± 0.93	43.60 ± 1.79	2.28 ± 3.36	Durowoju et al.^32^

*Nr* not reported.

### pH levels in groundwater of the study area

The pH of all the samples were in the range of 8.10–9.19 and complied with the South African (2014) National Standards for drinking water of 5.0 to 9.5. The samples with higher temperature (45–48 °C) in this study recorded slightly higher pH (8.86–9.10) than those with a lower temperature (25–27 °C) but this difference was not statistically significant (p > 0.05). Odiyo and Makungo^35^ reported pH levels in the range of 6.9–8.7 for groundwater around Siloam area. Durowoju et al.^32^ reported mean pH levels between 7.89 and 9.39 for geothermal springs in Siloam, Tshipise, Mpephu and Sagole areas of Northern Limpopo province. High groundwater pH is a proxy indicator of long residence time of groundwater-rock interaction^54,55^. Although pH affects fluoride mineralization differently, it is widely reported that alkaline pH favors the dissolution of fluoride from most fluoride bearing minerals^9,25^. Previous results on the monitoring of fluoride in the groundwater of Vhembe district in Limpopo Province shows the prevalence of alkaline pH^26,32^. In this study, high fluoride levels were also associated with alkaline pH values. A Pearson’s correlation analysis performed on the data in Table 2 between fluoride and pH yielded a positive correlation with R^2^ = 0.72. This shows that alkaline pH could have contributed to the mineralization of fluoride from their host rocks.

### Temperature levels in groundwater of the study area

The mean temperature ranged from 25 to 48 °C. Generally, temperature has a direct effect on chemical reactions as it speeds up the rate of chemical reaction and dissolution. Fluoride concentration was higher in hot water aquifer (H1 and H2) than in cold water aquifer (BH1 and BH2). At H1 and H2, the temperature of the groundwater was 45 °C and 48 °C yielding fluoride concentrations of 4.55 mg/L and 4.95 mg/L, respectively, whereas lower levels of fluoride (4.50 and 3.92 mg/L) were determined at groundwater temperatures of 27 °C and 25 °C, respectively. The temperature change of 23 °C resulted in 1.03 mg/L increase in fluoride concentration in groundwater. This is a significant increase in fluoride concentration. A similar trend was reported in Ethiopia by Tekle-Haimanot et al.^56^, where 80% of the groundwater samples obtained in the geothermal springs of the rift valley area contained fluoride concentrations above 3.0 mg/L and 30% contained fluoride concentrations above 13.0 mg/L compared to the cold springs in the same area with maximum fluoride concentration of < 3.0 mg/L. High geothermal temperature causes an increased dissolution of fluoride bearing minerals and thus increases the fluoride concentration in groundwater^35^. However, existence of geothermal springs does not necessarily mean fluoride will always be high. Therefore, water chemistry and local geology also plays an important role in the occurrence of high groundwater fluoride in any area. A positive correlation (R^2^ = 0.6) was computed between fluoride and temperature from the data presented in Table 2.

Previous studies conducted within the region have also reported varying temperatures. Temperature in the range of 26.9–63.86 °C have been reported for boreholes in Siloam area^35^. Durowoju et al.^32^ reported temperatures between 41.3–67.7 in thermal springs around the study area. Kirkpatrick^57^ reported higher fluoride levels in Malawi which were associated with higher geothermal temperature. His report shows that higher fluoride levels (17 mg/L and 20 mg/L) were determined at 65 and 79 °C when compared to 3–12 mg/L recorded within the temperature range of 32–54 °C. Results from several other studies agrees that geothermal temperature generally leads to more fluoride mineralization in groundwater^14,23^.

### Influence of calcium on fluoride levels in groundwater

In this study, Ca varied differently in the sites (Table 2). Generally, the sites H1 and H2 with the lowest Ca concentration recorded the highest fluoride level. Although BH2 recorded lower Ca levels, the fluoride level was lower than that of BH1. It has been widely reported that low calcium concentration in groundwater often increases the dissolution of CaF_2_ which increases fluoride levels in groundwater^26,33^. Chae et al.^58^ stated that there is a thermodynamic relationship between Ca^2+^ and F^-^ ions which is controlled by the equilibrium of fluorite. Odiyo and Makungo^35^ reported an inverse relationship between fluoride and calcium levels in groundwater around Siloam. A negative correlation (R^2^ = − 0.30) was computed between fluoride and calcium levels in the groundwater of the study area reported in Table 2.

Nezli et al.^59^ however reported that when there is groundwater saturation with respect to fluorite, low calcium levels in groundwater can yield high fluoride concentrations. Odiyo and Makungo^33^ reported a mean level of calcium in the range of 0.38–1.44 mg/L associated with high levels of fluoride (1.34–6.74 mg/L) from 10 borehole samples from Siloam area. The chemistry of the groundwater at Siloam partially agrees with the opposing factor of solubility between calcium and fluoride because it appears that geothermal temperature plays an important role in the inverse solubility trend between fluoride and calcium.

### Sulphate, nitrate and electrical conductivity levels in groundwater samples

The levels of sulphate recorded in this study complied with the regulatory standards for drinking water. Similarly, the levels of sodium, potassium, chloride all complied with regulatory standards (Table 3).

**Table 3:** Chemical parameters of groundwater.

Parameters	Sampling sites				WHO (2011)
(mg/L)	H1	H2	BH1	BH2	
NO_3_^−^	0.17	1.31	3.22	83.95	44.0
Cl^−^	153.30	38.90	80.14	103.23	250
SO_4_^2−^	16.45	10.55	17.56	25.88	500
PO_4_^3−^	1.15	1.52	3.29	4.59	N/A
CO_3_^2−^	1.80	2.40	0.00	2.70	20.0
HCO_2_^−^	8.54	9.76	31.11	15.86	N/A
Na^+^	118.00	62.70	124.00	170.00	400
K^+^	2.73	2.21	5.15	4.67	400
Mg^2+^	Bdl	Bdl	15.80	3.72	100
TDS	306.00	130.00	296.00	423.00	450.00
EC (µS/m)	63.00	33.00	69.00	73.00	150.00

STD *N/A* Not available, *H1 & H2* individual borehole 1 & 2, *BH1* & *BH2* monitoring borehole 1 & 2, *Bdl* Below detection limit.

Nitrate contamination is a growing problem and ingestion by infants can cause methemoglobinemia and death^60,61^. High concentration of nitrate in groundwater above the permissible limit of 44 mg/L is considered an indicator of anthropogenic pollution^62^. Nitrate source is mostly from septic tanks, pit latrines and fertilizers. The water table in well BH2 occurs at 28 m below the surface, and this makes it easy for infiltration of sewage from nearby pit latrines located less than 20 m uphill from the well. The nitrate levels of the other boreholes complied with the regulatory guideline limit.

The electrical conductivity (EC) and total dissolved solids (TDS) values for all the locations sampled within Siloam were within the recommended limits of < 150 µS/m and < 450 mg/L, respectively^52^.

### Geochemical classification of groundwater

**Figure 3:**
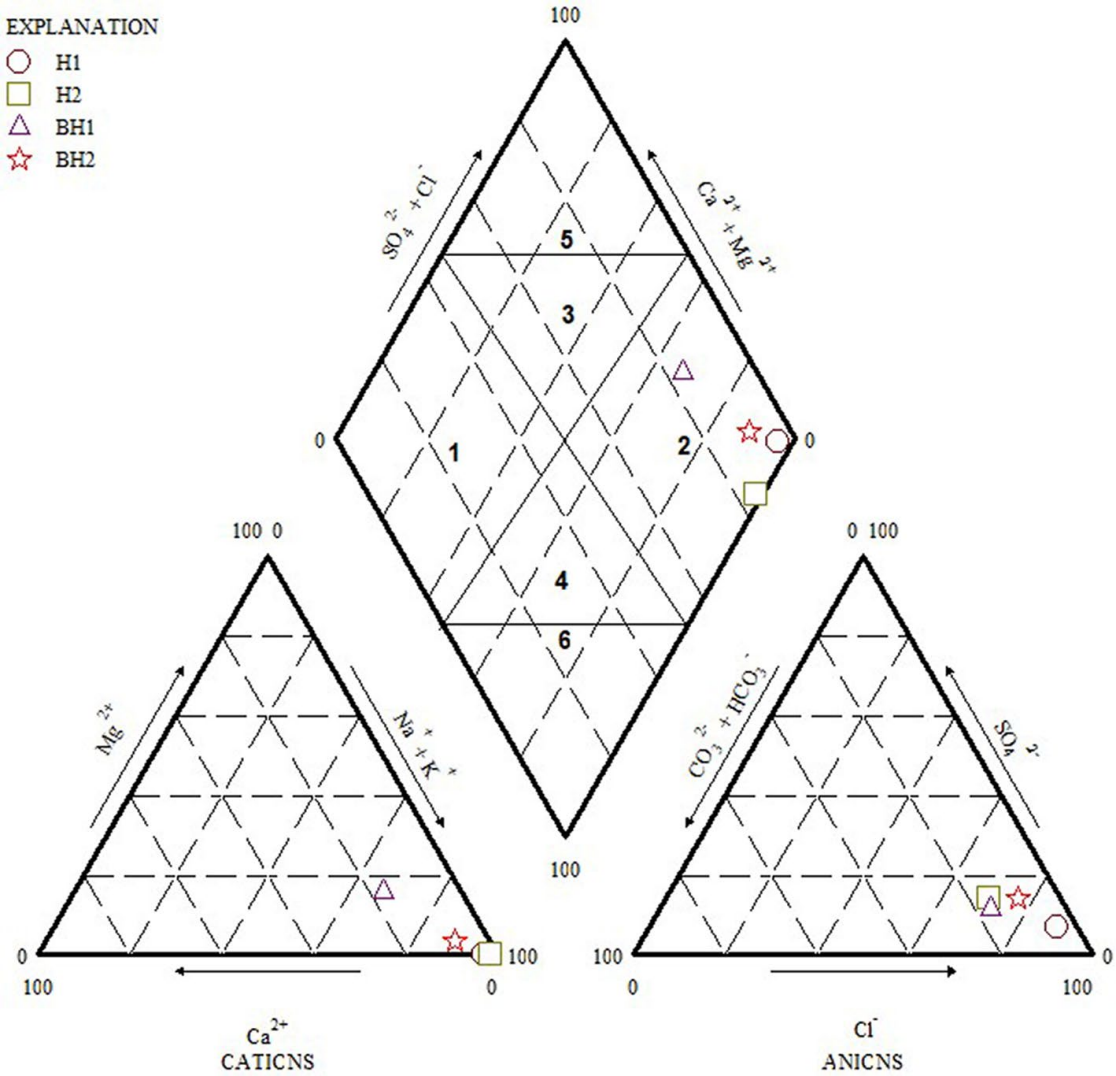
Piper Trilinear diagram of major anions and cations in Siloam groundwater (Figure drawn using GW Chart version 1.30: U.S. Geological Survey Software Release, 26 June 2020, https://doi.org/10.5066/P9Y29U1H).

Groundwater chemistry is directly linked to its quality therefore it is imperative to understand the complex geochemical processes occurring below the surface of the earth through different geochemical facies present. The Major cations (Ca^2+^, Mg^2+^, Na^+^ and K^+^) were plotted against major anions (SO_4_^2-^, HCO_3_^-^ and Cl^-^) on a piper trilinear diagram, to understand the water type dominant in the groundwater of Siloam (Fig. 3).

Piper diagram divides water into six basic types based on the dominating cation and anion. The divisions are: (1) Ca-HCO_3_ type, (2) Na-Cl type, (3) Ca–Mg–Cl type, (4) Ca-Na-HCO_3_ type, (5) Ca–Cl type, and (6) Na-HCO_3_ type^63^. From the piper diagram (Fig. 3), all the samples were plotted within the sodium chloride (Na-Cl) quadrant. Na-Cl water type is dominated by Na^+^ and Cl^-^ derived from Na-Cl which could be linked to the underlying geology emanating from gneissic rocks. This water type is typical of marine and deeper ancient groundwater influenced by ion exchange. Although exceptions can be made in situations where dissolution factors like residence time, climate and rainfall, are not favorable for fluoride migration from the rock to groundwater as experienced in Eswatini Kingdom^32^.

A previous study based on the water type of thermal groundwaters around Siloam area indicated that the dominant water type is Na-Cl^26,32^. Groundwater mixing from different sources do not occur in the study area because the water is predominantly and 100% Na^+^–Cl^−^ type. A piper plot by Durowoju^64^ shows that the geothermal springs of Siloam in both wet and dry periods were dominated by Na-Cl ions. Therefore, seasonal changes do not significantly impact on the groundwater type and dominant cation and anions. However, groundwater with high fluoride in Korea occurs mostly within the basement aquifer and are dominantly Na-HCO_3_ water type influenced by granitoids and metamorphic rocks^58^. Elevated Na^+^ in groundwater could be from the weathering and dissolution of plagioclase tectosilicate mineral end member (albite). Na–Cl water type indicates ancient groundwater with ample residence time with associated overburden and aquifer material.

**Figure 4:**
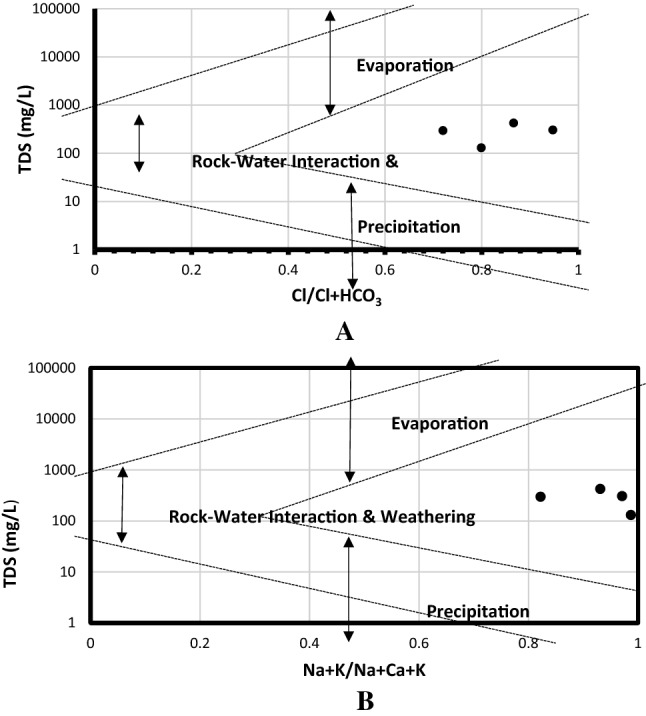
Gibbs plot showing dominant anion (**A**) and cation (**B**) mineralisation process of groundwater in Siloam.

Gibbs diagram was used to further buttress the source of the Na enrichment. The Gibbs diagram gave a clear indication that the groundwater enrichment was as a result of rock-water interaction and weathering (Fig. 4). The total dissolved solids were plotted against major anions and cations on Gibbs diagram to determine the geochemical process responsible for the chemical constituents of groundwater at Siloam using the Eqs. (1) and (2)^65^.

1 $${\text{Anions}} = \frac{{{\text{Cl}}}}{{{\text{Cl}} + {\text{HCO}}_{3} }}$$

2 $${\text{Cations}} = \frac{{{\text{Na}} + {\text{K}}}}{{{\text{Na}} + {\text{K}} + {\text{Ca}}}}$$

However, the occurrence of Cl^-^ > Na^+^ hydro-chemical process in borehole H1 (Table 2) denotes that although the prevailing water type is Na^+^—Cl^-^, the dominance of the water type is as a result of ion exchange and reverse ion exchange. The occurrence of sulphate (SO_4_^2-^) in groundwater is mostly assumed to be influenced by the dissolution of gypsum or the neutralization of acid water by limestone or dolomite^66^. Gibbs plot showed the geological process responsible for the mineralization process in the groundwater at Siloam.

**Figure 5:**
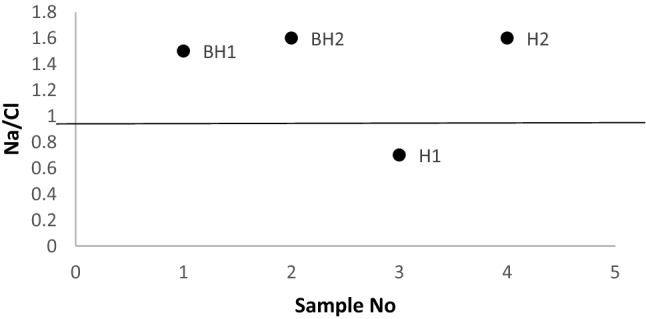
Plot showing Na/Cl ratio.

The plot of Na/Cl (Fig. 5) is a good indicator of the type of weathering responsible for the dominance of Na in groundwater. Mayback^67^ noted that Na/Cl ratio greater than one is an indication of silicate weathering, otherwise the source of Na is from another source other than silicate weathering. From the Na/Cl ratio plot, 75% of the samples (BH2, BH1 and H2) show predominant silicate weathering while 25% (H1) of the samples show other sources of Na^+^ other than silicate weathering. This other source is the reverse ion exchange. The dominant Na^+^ in the groundwater at Siloam is thus as a result of the weathering and chemical alteration of tectosilicate minerals in the form of Plagioclase feldspar (Na rich feldspar) or sheet silicate minerals in the form of chlorite, due to rock-water interaction over a period resulting in base ion exchange between the rocks, soil and groundwater.

**Figure 6:**
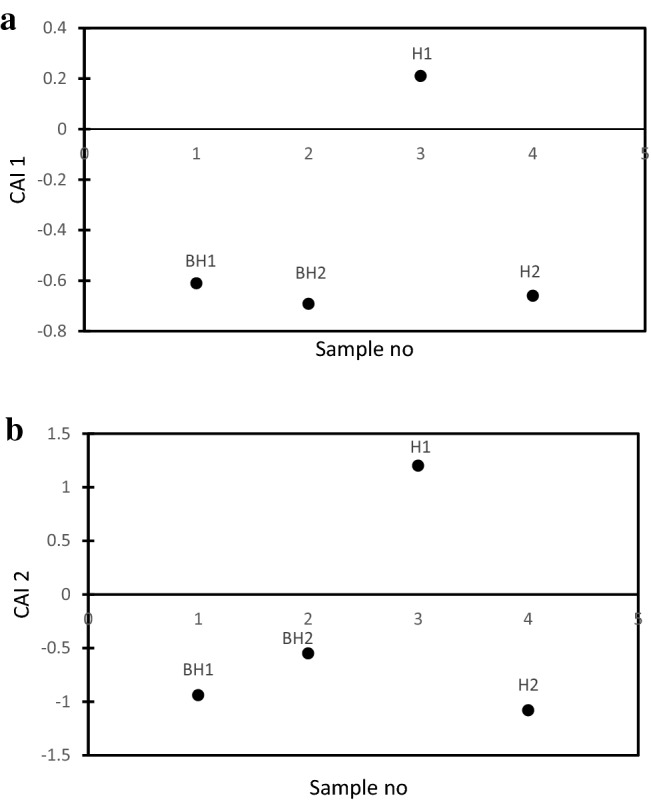
Chloro-Alkaline Index CAI 1 (**a**) and CAI 2 (**b**) for enrichment process.

Chloro-Alkaline Indexes (CAI) were calculated from Eqs. (3) and (4) to determine if the Na^+^ is from base ion exchange or from reverse ion exchange. In ion exchange process, the Ca and Mg from the groundwater is exchanged with the Na^+^ and K^+^ from the host rock or surrounding rocks. CAI 1 and CAI 2 were plotted in Fig. 6a,b.

3 $${\text{CAI~1 = }}\frac{{\left[ {{\text{Cl}}^{ - } \left( {{\text{Na}}^{{\text{ + }}} {\text{ + K}}^{{\text{ + }}} } \right)} \right]}}{{{\text{Cl}}}}$$

4 $${\text{CAI~2 = }}\frac{{{\text{[Cl}}^{ - } - \left( {{\text{Na}}^{{\text{ + }}} {\text{ + K}}^{{\text{ + }}} } \right){\text{]}}}}{{{\text{SO}}_{{\text{4}}} ^{{2 - }} {\text{ + HCO}}^{ - } _{{\text{3}}} {\text{ + CO}}_{3} ^{{2 - }} {\text{ + NO}}_{3} ^{ - } }}$$

CAI 1 and CAI 2 will result in a negative value if the process of Na^+^ enrichment is by ion exchange process, otherwise, the process of reverse ion exchange is responsible^68^. CAI 1 and CAI 2 from Fig. 6 shows that the boreholes BH1, BH2 and H2 exhibited a sodium enrichment process by normal ion exchange while borehole H1 shows that the sodium enrichment process is by reverse ion exchange process.

### Lithology and mineral composition of soils and rocks in the study area

The lithologic description of the samples is given in Table 4. Chlorite minerals can be found in any major rock group, but they are known to be predominant in low grade metamorphic rocks. Chlorite is also the index mineral for green schist facie^69^. Chlorite is found as a common constituent of igneous rocks resulting from hydrothermal alteration of pyroxenes, amphiboles, biotite and garnet^69^. In sedimentary basins, Clay-rich sediments derived from the weathering of igneous rocks are often rich in chlorite^69^. This is basically the source of chlorite enrichment in sedimentary terrain.

**Table 4:** Lithologic Description of Sampled Rocks and Soils.

Location	Sample ID	Depth (M)	Associated group	Lithology	Brief description
**S**	S1		Soil	Clay	Brown color with very fine texture
	SR2		Metamorphic	Epitomized grey wacke	This rock type shows a low-grade metamorphism characteristic and it exhibits an aphanitic texture. It has a green colour due to the abundance of chlorite
	SR3		Sedimentary	Sandstone	Pinkish Sandstone due to the presence of hematite
**BH1**	X5	5	Sedimentary	Argillaceous Sediment	Topsoil with brittle texture and pinkish-brown color. Presence of Smectite and hematite noticeable
	X15	15	Sedimentary	Argillaceous Sediment	Dark brown color with reduced pinkness probably due to the hematite content
	X20	20	Sedimentary	Argillaceous Sediment	Light pink color due to the heavy presence of clay mineral Smectite and Hematite
	X30	30	Sedimentary	Argillaceous Sediment	Reduction in the pink color and traces of darker brown color. Smectite and Hematite still present although at a reduced concentration
	X40	40	Sedimentary	Gray Wacke	Green in color probably due to the presence of Chlorite mineral and the rock is slightly saturated due to its function being the aquifer rock. Total disappearance of clay minerals of smectite and hematite
**BH2**	X65	65	Sedimentary	Gray Wacke	Green in color probably due to the presence of Chlorite mineral and the rock is slightly saturated due to its function being the aquifer rock. Total disappearance of clay minerals of smectite and hematite noticeable. Index mineral noticed are quartz and chlorite
	Y5	5	Soil	Clay	Light brown in color with more than 60 wt.% Smectite
	Y10	10	Soil	Clay	Light brown color with increased smectite content of more than 70 wt.%
	Y20	20	Soil	Clay	Reduced lightness with two different groups of Smectite namely smectite 1 and 2. Overall smectite content is greater than 75 wt.%
	Y25	25	Soil	Sandy Clay	Dark brown color with intercalation of clay and sand. Although smectite content is still above 50% but there is reduction in its wt.%
	Y28	28	Igneous	Basalt	Dark color with dominantly plagioclase feldspar and diopside mineral
	Y40	40	Igneous	Basalt	Dark color with dominantly plagioclase feldspar and diopside mineral

*S* surface soil, *SR* surface rocks, *BH1* borehole 1, *BH2* borehole 2.

The abundance of chlorite can be seen in Siloam which is made up of volcano sedimentary sequence. Table 5 gives the weight percent (wt.%) and the constituent minerals in all the soil and rock samples collected in Siloam as analysed by X-ray Diffraction (XRD). The abundance of chlorite is seen especially in borehole cuttings of BH1 at depths 40–65 m where chlorite is seen as much as 38 wt.% of the total rock mass at some depths (Table 5). However, the occurrence of chlorite determines the colour of the total rock mass given that the wt.% of chlorite is more than 20% as seen in cuttings X40 and X65 (Table 5). From depths 5–35 m in borehole BH1, the pink–brown colour could be due to the presence of smectite and occurrence of hematite. The brown colour may also be due to the presence of plagioclase.

**Table 5:** Mineral constituents and percentage weight of rocks and soils.

	S1	SR2	SR3	X5	X15	X20	X30	X40	X65	Y5	Y10	Y20	Y25	Y28	Y40
	Weight (%)														
Diopside	2.76	20.5												28.7	29.87
Chlorite		28.41		4.21	5.42	4.59	5.42	38.92	26.98					4.69	4.72
Hematite	1.7	0.17	0.67	9.41	7.87	5.07	7.27				0.78				
Muscovite			1.6	32.41	33.77	33.07	41.15	12.33	7.49					4.56	3.29
Orthoclase	8.04			2.65	1.87					7.7	5.61	7.97	14.99		
Plagioclase	32.38	14.82						17.35	16.31	11.08	6.86	9.75	22.93	39.49	38.27
Quartz	8.36	14.47	97.73	35.99	34.44	44.76	30.04	25.99	20.72	12.19	8.34	5.53	9.72	7.69	9.00
Smectite	44.26			15.33	16.63	12.51	16.1			69.02	78.41		52.36	5.88	7.1
Smectite1												41.2			
Smectite 2												35.55			
Sepiolite	2.49														
Epidote		21.63							12.04						
Titanite								5.4	7.25						
Actinolite									9.22					2.08	2.6
Enstatite														3.02	
Ilmenite														3.89	5.18

*S* surface soil, *SR* surface rock, *X* borehole BH1 depth, *Y* borehole BH2 depth

However, occurrences of quartz and muscovite mostly do not determine the colour of the whole rock mass because they are mostly colourless to white in colour. The pinkish colour of rock SR2 could signify the occurrence of hematite (iron oxide). The occurrence of chlorite in the sedimentary terrains of Siloam and the high abundance of smectite and hematite group shows that the present geologic sequence has undergone a series of weathering and tectonic activities. This explains the abundance of Na^+^ in groundwater at Siloam because of ion exchange and possible reverse ion exchange during the process of weathering of silicate minerals like plagioclase and chlorites.

From the mineralogy (Table 5), silicate minerals as well as clay minerals are abundant in the study area. The area is dominated by clay minerals such as chlorite and smectite, as well as silicate minerals such as muscovite, biotite and plagioclase. Clay can adsorb and desorb fluoride under favorable condition. Clay in the soil can functions as a natural barrier to protect groundwater from fluoride pollution due to its strong adsorption potential^70^. The adsorption capabilities of clay are possible in the acidic pH conditions. However, the adsorbed fluoride ion can also be released into the groundwater under alkaline conditions.

The adsorption properties of fluoride by clay are proportional to the amount of clay minerals present. Clay desorption is a faster geochemical process when compared to adsorption process^71^, thus it is easy for fluoride to migrate from clay to groundwater when in contact with small volume of alkaline water. The abundance of clay minerals show that extensive weathering altered the chemical and physical composition of the silicate minerals to form clay minerals. The fluoride enrichment of the groundwater in the area is as a result of ion exchange between the fluoride bearing minerals and groundwater. However, muscovite also contains fluoride as an accessory ion in its chemistry, which at favorable conditions, is released into the groundwater.

Muscovite is an important fluoride bearing mineral because it contains fluorine in its crystal lattices and hydroxyl groups which can also substitute for fluoride because of their similar ionic charges^70^. This fluorine can be released as fluoride ion into the groundwater under favorable conditions. Muscovite occurs at about 1.62 wt. % (Table 5) in SR3. Onipe^48^ noted that muscovite in the surface rocks at Siloam contributes to the groundwater fluoride rather than fluorite as earlier inferred by McCaffrey and Willis^25^ and Odiyo and Makungo^35^. Onipe^48^ also argued that the muscovite present in the surface sandstone at Siloam can never be the sole contributor of fluoride to groundwater of the area.

**Figure 7:**
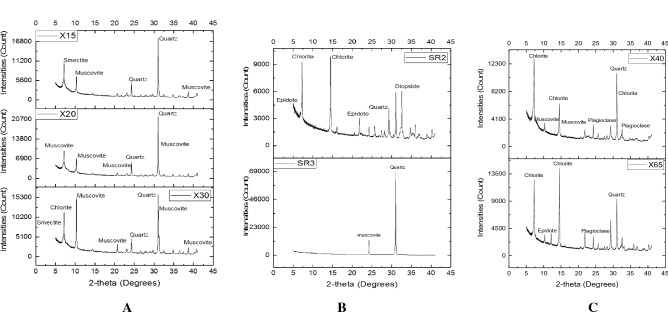
XRD plot showing major minerals in borehole 1 at X15, X20 & X30 (**A**) and surface soil SR2 & SR3 (**B**) borehole 1 at X40 and X65.

Surface rock SR2 contains high proportions of chlorite and epidote (Fig. 6). The occurrence of clinochlore, an end member of chlorite is predominant in Siloam. Chlorite has been linked to fluoride contribution to groundwater mostly due to its hydroxyl content which is displaced by fluoride ion. Generally, chlorite contains fluoride but not as much as its ripidolite. This reason is attributed to the fact that clinochlore has an appreciable number of aluminium in its octahedral layer therefore limiting its fluoride adsorption properties unlike ripidolite that has aluminium in its tetrahedral layer. However, at varying depth in borehole BH1, muscovite occurs as an abundant rock forming mineral rather than its accessory form as found in SR2. The concentration of muscovite in wt. % increases from topsoil to 30 m below the surface (Table 5). The major minerals present in borehole BH1 from depth 5 m to 30 m are muscovite, quartz and smectite (Table 5). The abundance of major dominating minerals of borehole BH1 from depth 15–30 m are shown in Fig. 7. Fluorine affiliated muscovite increases with depth from 5 to 15 m, slows down at depth 20 m and picks up its increasing trend again at depth 30 m below ground level before drastically reducing as the depth increases.

Figure 7C shows the XRD peaks for the epitomized gray-wacke which is the aquifer rock for borehole BH1. Although muscovite is present, the abundance is not as much as at depths 5 to 30 m. The grey wacke is dominated by chlorite which explains why the color is greenish grey (Table 4). The primary minerals present in the rock materials include chlorite, quartz and plagioclase. The intensities and abundance of each mineral are identified in Fig. 7. The combined presence of muscovite and chlorite at depth X40 and X 65 in borehole BH1 may accounts for the slightly high total fluoride concentration of the aquifer due to the exchange of OH with F in their structure. Figure 7 also shows a high abundance of plagioclase which denotes that the level of weathering is either absent or low at depth 40 m below the earth surface, therefore there has been no chemical alteration of plagioclase to smectite clay or kaolin as seen at shallow depths before 40 m. Two main dynamic geochemical processes occur in clay, they are the enrichment and leaching processes. Under the dynamic geochemical process of leaching, areas with clay formations and arid to semi-arid climatic conditions, experience high groundwater and surface water fluoride concentration^72^. Dynamic geochemical process of leaching occurs when fluoride is leached out of clay formation into groundwater through infiltration and percolation of water. Fluorine can easily migrate from clay formations to groundwater during migration of groundwater^73^.

In borehole BH2 at Siloam, muscovite which is the predominant fluoride bearing mineral in the area is seen to be absent until the depth of 28 m (Table 5). Muscovite occurrence was found at depth 28 to 40 m although at a volume less than 5 wt.%. The peaks of smectite at depths Y5, Y10, Y15 and Y20 occur at almost the same count and intensity (Fig. 7). Smectite is the most abundant mineral at depths Y5–Y20. Clay could also act as a migration agent for fluoride enrichment of groundwater at Siloam. The abundance of major minerals in borehole BH2 at depths Y28 and Y40 are also described in Table 5. The abundance of chlorite and plagioclase were also revealed from the results. Y28 and Y40 is the basaltic aquifer material of borehole BH2.

### Chemical composition of rocks and soils in the study area

**Figure 8:**
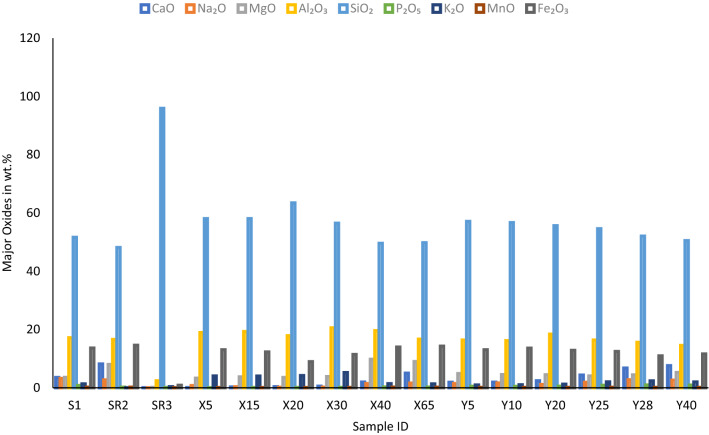
Major oxide abundance in sampled rocks and soils.

In sedimentary terrain, the most abundant fluorine bearing mineral is associated with mica mineral group and clay minerals especially montmorillonite and kaolinite. Total rock chemistry is important to determine the total rock composition in oxides and elemental properties. The graphical representation of major oxides in the rocks and soil of Siloam are presented in Fig. 8. The rocks and soil are dominated by high silica content followed by aluminium oxides. The highest concentration of silica occurs at surface sandstone rock of location SR3. The formula for conversion of oxides to elemental weight. percent is given in Eq. (5).

5 $${\text{Oxide~wt~percent~}} \times {\text{Conversion~factor~}} = {\text{~Elemental~wt~percent}}$$

The conversion factor is the sum of the atomic weights of the elements in the required formula divided by the sum of the atomic weights in the original formula. Silica (SiO_2_) and quartz (SiO_2_) have the same chemical composition, but they are different in nature. Silica refers to the total pure silicon dioxide in the rock mass while quartz refers to a mineral with a very high concentration of silica. However, often, quartz occurs as silica with little amount of impurities. Therefore, the name is used interchangeably. Total rock silica could be from quartz and other silicate minerals like feldspars, chlorites and other aluminium and silicate rich minerals. The SiO_2_ refers to the total silica content of the rock and soil. Silica has the highest wt. % in soils and rocks of Siloam. This means that the area is dominated by felsic rock and the soil with high silica content. Felsic rocks, which contain relatively high concentrations of SiO_2_, tend to have higher concentrations of fluorine than mafic rock^74^.

Table 6 shows the total major elemental and fluorine compositions of rocks and soils at Siloam in mg/kg. Although fluorine is regarded as a trace element, its concentration in some rocks and soil in Siloam is higher than expected. The total fluorine content of rocks and soil ranges from 10 to 2000 mg/kg (Table 6). The average fluorine concentration is about 762 mg/kg. This is above the permissible limit of 30 mg/kg concentration of fluorine in soil for agriculture^75^. The highest fluorine concentration of 2000 mg/kg occurs at a depth of 5 m in borehole BH2. The lowest concentration occurs in borehole BH1 at a depth of 30 m (Table 6).

**Table 6:** Major elemental compositions of rocks and soils (mg/kg) in the study area.

Sample ID	Ca	Na	Mg	Al	Si	P	K	Mn	Fe	F
S1	25,429.08	23,889.18	21,587.40	90,880.81	241,417.00	3500.73	11,206.35	1541.26	95,607.98	431.00
SR2	58,286.88	20,253.87	48,240.00	87,863.80	225,241.50	969.03	305.48	1657.43	101,972.52	1400.00
SR3	415.72	242.60	940.68	12,650.27	448,332.50	484.52	3137.78	41.82	6329.57	160.00
X5	658.58	5749.73	20,019.60	100,461.14	271,477.25	281.54	33,619.05	569.26	91,551.46	450.00
X15	2021.47	2559.56	22,672.80	102,101.97	271,617.50	471.42	33,536.04	723.38	86,096.14	330.00
X20	2521.48	1409.61	21,406.50	94,744.70	296,628.75	475.79	35,030.22	749.72	62,806.12	290.00
X30	4057.22	1468.96	23,215.50	108,877.01	264,231.00	659.12	43,580.25	836.46	79,941.42	10.00
X40	14,143.14	10,757.55	59,094.00	103,848.66	231,926.75	1169.82	11,621.40	1773.61	97,706.18	850.00
X65	36,000.72	11,944.59	54,330.30	88,340.17	232,815.00	999.59	11,123.34	1618.71	100,084.14	770.00
Y5	13,500.27	10,905.93	29,486.70	86,911.06	267,129.50	2330.91	8301.00	1184.99	91,551.46	2000.00
Y10	13,785.99	12,389.73	27,376.20	85,587.81	265,166.00	2099.57	8633.04	1239.20	95,188.34	1500.00
Y20	17,143.20	8457.66	26,954.10	97,444.13	260,163.75	2549.16	10,210.23	1215.97	89,872.90	1200.00
Y25	31,214.91	14,021.91	24,602.40	86,805.20	255,442.00	3727.71	17,017.05	1394.10	87,145.24	800.00
Y28	51,572.46	20,847.39	29,124.90	82,041.50	239,593.75	4932.45	19,922.40	1014.60	74,695.92	620.00
Y40	54,429.66	19,437.78	31,959.00	76,960.22	236,181.00	4234.05	16,851.03	1177.24	81,340.22	620.00

*S* surface soil, *SR* surface rock, *X* borehole BH1 depth, *Y* borehole BH2 depth, ion units, *mg/kg* depth: m.

The fluorine concentration in clay is typically in the range of 20–500 mg/kg^76^. The surface clay deposit S1 has a total fluorine concentration of 431 mg/kg (Table 6). However, the clay formation of borehole BH2 from 5 m to around 20 m depth has the highest concentrations of fluorine. The Soil of BH2 at depths 5 m and 10 m have more fluorine concentration than the surface rock formations and aquifer rocks in Siloam as observed from Table 6. This is different from the findings of McCaffrey and Willis^25^ that stated that rock samples have higher fluorine content than soil samples. The clay deposit and argillaceous sediments of Siloam are from chemical alteration and weathering of plagioclase of parent rock materials therefore giving more room for OH and F replacements. This ion displacement gives a higher room for more storage of fluoride in the crystal lattices of the resultant clay. It should be noted, however, that clays formed under hydrothermal conditions are mostly rich in fluoride^77^ as observed in Siloam, although this is not always the case as observed in Sagole, Tshipise and Evangelina area of Limpopo, South Africa^78^.

### Fluoride leaching and other chemical constituents from rocks and soils

**Figure 9:**
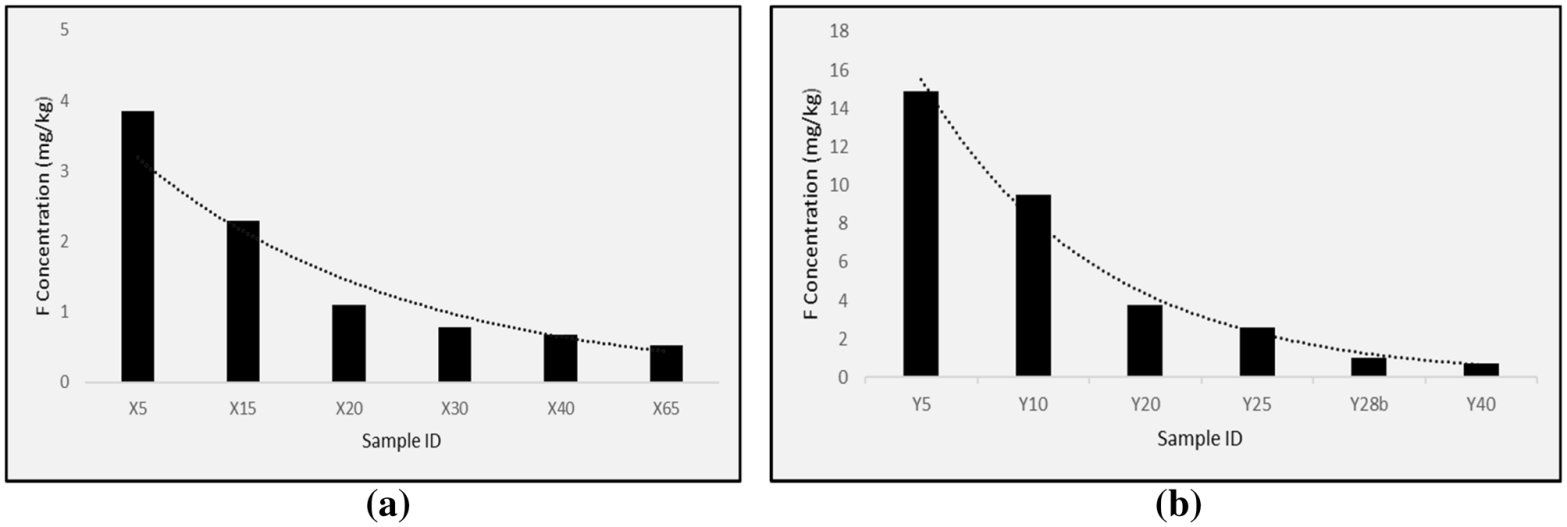
Fluoride concentration of (**a**) borehole 1 and (**b**) borehole 2.

The fluoride concentration emigrating out of the rock and soil samples of Siloam ranges from 0.27 to 14.88 mg/L (Table 7). Leachate with the lowest fluoride concentration occurs at surface rock SR2 and leachate with the highest fluoride concentration occurs at a depth of 5 m of borehole BH2 (Table 7). Fluoride concentrations show a decreasing trend from the surface down to the aquifer. Figure 9 shows the fluoride concentration trend in the borehole BH1 leachates.

**Table 7:** Chemical parameters in the leachate obtained from leaching experiment.

Sample	**K**	**EC**	**pH**	**Ca**	**Mg**	**Cl**	**NO**_**3**_	**NO**_**2**_	**PO**_**4**_	**Na**	**B**	**F**
	mg/kg	µs/cm		mg/kg	mg/kg	mg/kg	mg/kg	mg/kg	mg/kg	mg/kg	mg/kg	mg/kg
**S 1**	62.50	24.00	7.57	135.00	891.00	36,03	1,65	0.00	1,89	**929.00**	1.75	10,52
**SR 2**	11.60	3.00	7.17	BDL	40.00	2,75	0,12	0.00	0,67	36.10	0.05	0,27
**SR 3**	27.80	3.00	6.83	BDL	BDL	2,77	0,21	0.00	3,17	2.26	0.04	0,44
**X 5**	379.00	42.00	7.10	19.80	773.00	**114,43**	24,66	0.00	1,55	**1020.00**	2.07	3,85
**X 15**	125.00	7.00	6.91	2.26	172.00	10,59	2,41	0.00	0,78	110.00	0.46	2,29
**X 20**	118.00	3.00	6.95	1.88	150.00	2,15	0,60	0.00	0,65	52.40	0.28	1,10
**X 30**	164.00	3.00	7.03	11.90	183.00	1,61	0,72	0,03	0,59	35.90	0.38	0,78
**X 40**	33.40	4.00	7.16	18.20	179.00	1,43	0,58	0,13	0,30	72.00	0.26	0,68
**X 65**	31.90	9.00	7.37	36.30	41.20	2,34	0,56	0,71	0,50	82.40	0.02	0,53
**Y 5**	31.90	32.00	8.00	186.00	675.00	11,57	0,78	0.00	0,39	**886.00**	0.93	14,88
**Y 10**	45.60	76.00	7.97	145.00	438.00	**171,79**	8,10	0.00	1,00	**1620.00**	1.11	9,51
**Y 20**	26.00	6.00	7.16	68.00	178.00	2,12	0,85	0.00	0,39	129.00	0.26	3,79
**Y 28**	61.90	9.00	7.43	94.80	97.90	1,93	0,29	0,33	0,90	151.00	0.15	1,02
**Y 25**	25.30	5.00	7.10	19.30	61.60	1,59	0,15	0,01	0,70	84.20	0.14	2,60
**Y 40**	54.50	10.00	7.50	110.00	104.00	2,46	0,27	0,49	0,80	166.00	0.17	0,72

*BDL* Below detection Limit, *S* surface soil, *SR* surface rock, *X* borehole BH1 depth, *Y* borehole BH2 depth

The fact that from boreholes BH1 and BH2, the sub-surface rocks and soils of Siloam show decreasing trends in fluoride concentrations, means that the aquifer rocks release less fluorine into the groundwater compared to the regolith and overburden soils and clays. This may partially be due to the loose state of the overburden. The ions in the leachate were correlated against each other to determine the relationship between them in relation to the release of fluoride. Table 8 shows the correlation coefficients in the leachate at Siloam. Fluoride concentrations in groundwater of boreholes BH1 and BH2 show high positive correlations to pH, Ca, Na (Table 8).

**Table 8:** Correlation of physico-chemical constituent of leachates.

	*K*	*EC*	*pH*	*Ca*	*Mg*	*Cl*	*NO*_*3*_	*NO*_*2*_	*PO*_*4*_	*Na*	*B*	*F*
*K*	1.00											
*EC*	0.25	1.00										
*pH*	− 0.27	0.69	1.00									
*Ca*	− 0.26	0.60	0.94	1.00								
*Mg*	0.44	0.64	0.50	0.56	1.00							
*Cl*	0.41	0.94	0.44	0.33	0.55	1.00						
*NO*_*3*_	0.85	0.60	0.02	− 0.03	0.56	0.74	1.00					
*NO*_*2*_	− 0.20	− 0.19	0.17	0.09	− 0.35	− 0.24	− 0.21	1.00				
*PO*_*4*_	0.15	0.11	− 0.22	− 0.09	0.17	0.19	0.22	− 0.22	1.00			
*Na*	0.26	0.97	0.72	0.67	0.80	0.89	0.59	− 0.27	0.15	1.00		
*B*	0.62	0.70	0.36	0.39	0.95	0.70	0.76	− 0.37	0.27	0.82	1.00	
*F*	− 0.06	**0.67**	**0.77**	**0.82**	**0.80**	0.44	**0.16**	− 0.35	0.01	**0.79**	0.64	1.00

This means that at an increasing F concentration, pH, Ca and Na increases. The concentrations of these highlighted parameters (pH, Ca, Na) are directly proportional to each other. EC is also positively correlated to F, which means that increase in fluoride results in an increase in pH, Ca and Na which in turn increases the conductivity of the groundwater of boreholes BH1 and BH2. The very weak correlation between fluoride and nitrate confirms that the possible source of high nitrate in groundwater of BH2 is not from a natural source rather from anthropogenic sources which were previously inferred.

The weak negative correlation between fluoride and potassium connotes that most of the occurrence does not originate from K bearing minerals. The groundwater fluoride of the study area is controlled by the desorption and adsorption properties of clay and partly by chlorite. Therefore, the groundwater hydrochemistry is dominated by Na-Cl water type signifying the importance of Na weathering and dissolution to investigating groundwater fluoride source. The process is simplified by Eq. (6).

6 $${NaAlSi}{}\user2{O}_{8} + 2H^{ + } + 9H{}O \to Al{}Si{}O_{5} \left( {\user2{OH}} \right){} + 4H{}SiO{} + 2\user2{Na}$$

The hydroxyl ion in the clay is displaced by fluoride in the structure lattice of the kaolin-smectite clay due to the similarity in their charge and radius. The resultant Na in the equation explains the abundance of Na in groundwater at Siloam. The study area is dominated by rocks of high silica content (Table 6). The silica content is a combined value of all the silicate minerals (quartz, plagioclase and muscovite). Rocks with high silica content often have high fluorine concentration^74^. This agrees with this study because there seem to be a decreasing trend in the fluorine content towards the mafic rocks at depth. Also, a decreasing trend could be noticed as opposed to a general norm that fluoride increases with depth. This is possibly due to the clay and argillaceous overburden. At BH1 and BH2, fluoride decreases from depth X5 to X65 and Y5 to Y40. This coincides with the decreasing concentration of smectite and chlorite in the mineralogy of the rocks.

### Effect of temperature on fluoride release from soil and rocks

**Figure 10:**
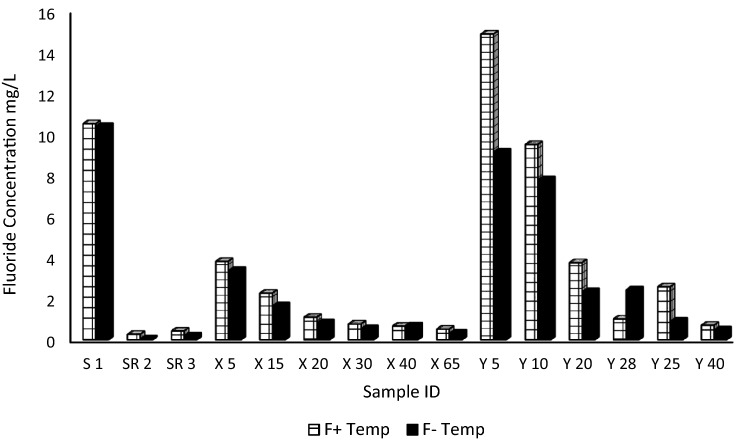
Fluoride leachate behaviour at room temperature and at 40 °C, F + Temp = Fluoride concentration at induced temperature and F-Temp = fluoride at concentration at room temperature.

Siloam is located on Siloam fault which acts as a channel for transport of heated groundwater up to some near surface aquifer. This gives Siloam a very high geothermal gradient. Therefore, the effect of temperature is very important when investigating the source of fluoride from the dissolution of minerals irrespective of the temperature of the water at present. The effect of temperature in the dissolution of fluoride from the mineral constituents in the rocks and soil into the groundwater was experimented in the laboratory. The average water temperature of 42 °C from past literature focusing on Siloam was used as the base temperature. Although, the effect of temperature was simulated for less than 10 h. Temperature plays an important role by increasing the fluoride concentration in the leachates. This difference can be observed in Fig. 10 where the comparison can be seen between fluoride release at room temperature and fluoride release at 40 °C.

The temperature effect can be seen at almost all sampling points except for Y28 (Fig. 10). The sampling point Y5 shows a distinct effect of temperature by a concentration difference of almost 5 mg/L. The groundwater temperature is evident to be one of the driving factors favoring the dissolution of fluorine from the rocks and soils at Siloam, into the groundwater in the area.

## Conclusions

Fluoride levels exceeding 1.5 mg/L recommended by the WHO was determined in the groundwater of the study area. The level is in the range that could cause both fluorosis and non-fluorosis diseases to the consumer of the groundwater resource. Positive correlation was determined between fluoride levels with pH and temperature while a negative correlation was determined with calcium. The major water type present in the study area is the NaCl water type. The geological process responsible for the enrichment of groundwater is rock-water interaction and weathering. However, chemical processes activated by the rock-water interaction were identified as ion exchange and reverse ion exchange. The host minerals determined from this study are the muscovite, chlorite and smectite. The study also revealed a decreasing trend in the fluoride concentrations from the surface down to the aquifer. From the hydrogeochemical analysis, a final conclusion can be drawn that muscovite, smectite and chlorite minerals are the main contributor of fluoride to groundwater in the study area. Groundwater temperature was also determined as a major driving force for fluoride dissolution.

## Data Availability

The data used in this study has been included in the manuscript.
